# Synthesis of *meso-*substituted dihydro-1,3-oxazinoporphyrins

**DOI:** 10.3762/bjoc.9.53

**Published:** 2013-03-07

**Authors:** Satyasheel Sharma, Mahendra Nath

**Affiliations:** 1 Department of Chemistry, University of Delhi, Delhi 110 007, India

**Keywords:** dihydro-1,3-oxazinoporphyrins, iminoporphyrins, La(OTf)_3_, Mannich type reaction, synthesis

## Abstract

Novel dihydro-1,3-oxazinoporphyrins and naphtho[*e*]bis(dihydro-1,3-oxazinoporphyrin) derivatives, in which the porphyrin macrocycle is covalently linked to the dihydro-1,3-oxazine ring system were successfully synthesized from 5-(4-aminophenyl)-10,15,20-triphenylporphyrin in good yields. The structures of the target products were established on the basis of spectral data and elemental analyses.

## Introduction

Porphyrin macrocycles are of crucial interest for their potential applications in diverse fields such as biomimetic models for photosynthesis [1–2], electronic materials [3], catalysis [4] and medicine [5–6]. In the past few decades, the synthesis of porphyrin derivatives has emerged as one of the major areas of research due to the success of these molecules for the eradication of malignant cells by photodynamic therapy (PDT) after their selective accumulation [7–10] in neoplastic tissues. In addition, the low dark-toxicity profile, easy removal from the tissue, and efficiency in generating reactive oxygen species by the absorption of photons in the visible or near IR region make them ideal candidates for developing effective photodynamic agents. These findings have encouraged researchers to design and synthesize potential targeting anticancer drugs derived from porphyrins [11–12]. Previously, a large number of these molecules have been synthesized by the coupling of diverse pharmaceutically important moieties, such as carbohydrates [13–15], amino acid residues [16–19], steroids [20–21], glycosides [22–24], nitroxyl derivatives [25], pyrrolidinone [26], pyrrolidine [27] and piperazine [28], to the porphyrin periphery. In addition, many porphyrin dimers and trimers have displayed significant biological efficacy [29] and some of these are used as photosensitizers in PDT applications for the treatment of various types of cancers [30].

Thorough literature search revealed that heterocycles containing a dihydro-1,3-oxazine ring system exhibit a wide spectrum of pharmacological activities, for example, acting as antimicrobial [31–33], anti-HIV [34], antimalarial [35] or antitumor agents [36–37]. By considering the anticancer significance of these two classes of molecules, it was contemplated to construct new dihydro-1,3-oxazinoporphyrins combining the porphyrin and dihydro-1,3-oxazine moieties in a single molecular framework. Such hybrid compounds may prove useful for pharmacological studies or in the development of new phototherapeutic agents. Therefore, in continuation of our efforts towards the synthesis of diverse porphyrin analogues [38–41] through peripheral functionalization of easily accessible *meso*-tetraarylporphyrins, we now report herein the first synthesis and spectroscopic characterization of a novel series of dihydro-1,3-oxazinoporphyrins.

## Results and Discussion

**Scheme 1:**
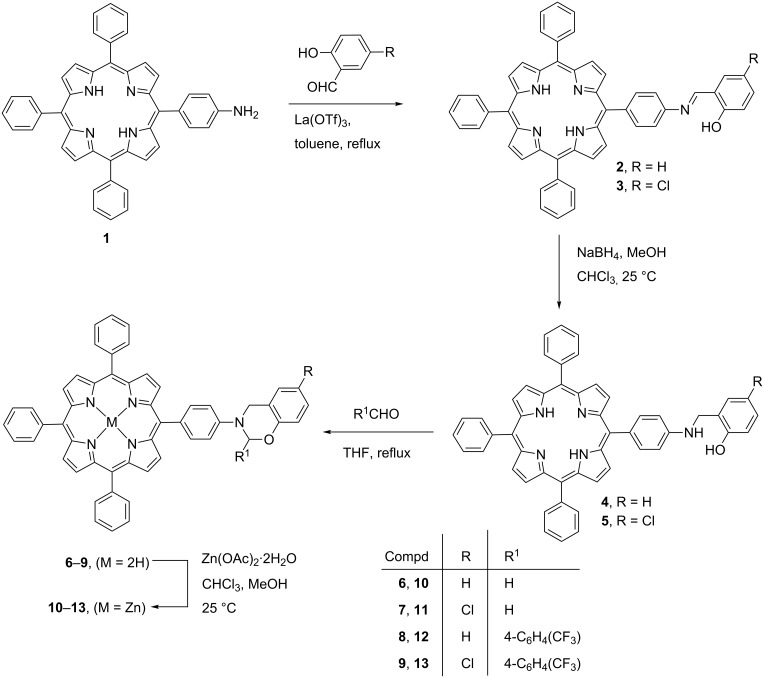
Synthesis of dihydro-1,3-benzoxazinoporphyrins.

The targeted dihydro-1,3-benzoxazinoporphyrins **6–9** were prepared in a three step procedure, starting from 5-(4-aminophenyl)-10,15,20-triphenylporphyrin (**1**), which was obtained by the reduction of 5-(4-nitrophenyl)-10,15,20-triphenylporphyrin using SnCl_2_ under acidic conditions [41–42]. Firstly, *meso-*(4-aminophenyl)porphyrin **1** was reacted with salicylaldehyde or 5-chlorosalicylaldehyde in the presence of La(OTf)_3_ as a Lewis acid catalyst in toluene under reflux to afford the corresponding iminoporphyrins **2** and **3**, which on reduction by NaBH_4_ in a chloroform/methanol mixture at 25 °C produced *meso-*substituted aminoporphyrins **4** and **5**, respectively. In the final step, these aminoporphyrins underwent a condensation cyclization reaction with aldehydes in THF under reflux to form new dihydro-1,3-benzoxazinoporphyrins **6–9** in good yields. Further, these free-base porphyrins were successfully converted to their zinc(II) analogues **10–13** by using Zn(OAc)_2_·2H_2_O as outlined in Scheme 1.

**Scheme 2:**
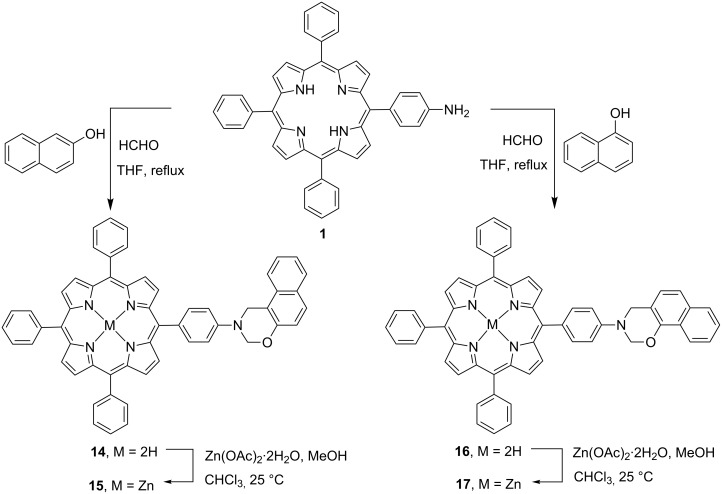
Synthesis of dihydro-1,3-naphthoxazinoporphyrins.

In contrast, the synthesis of new dihydro-1,3-naphthoxazinoporphyrins **14** and **16** was achieved in 75–85% yields in a one-pot three-component Mannich type condensation–cyclization reaction of 5-(4-aminophenyl)-10,15,20-triphenylporphyrin (**1**) with α- or β-naphthol and formaldehyde in THF under reflux. After reaction with Zn(OAc)_2_·2H_2_O in CHCl_3_/MeOH mixture, the free-base naphthoxazinoporphyrins **14** and **16** underwent zinc insertion to afford zinc (II) dihydro-1,3-naphthoxazinoporphyrins **15** and **17** in 90–92% yields as depicted in Scheme 2.

**Scheme 3:**
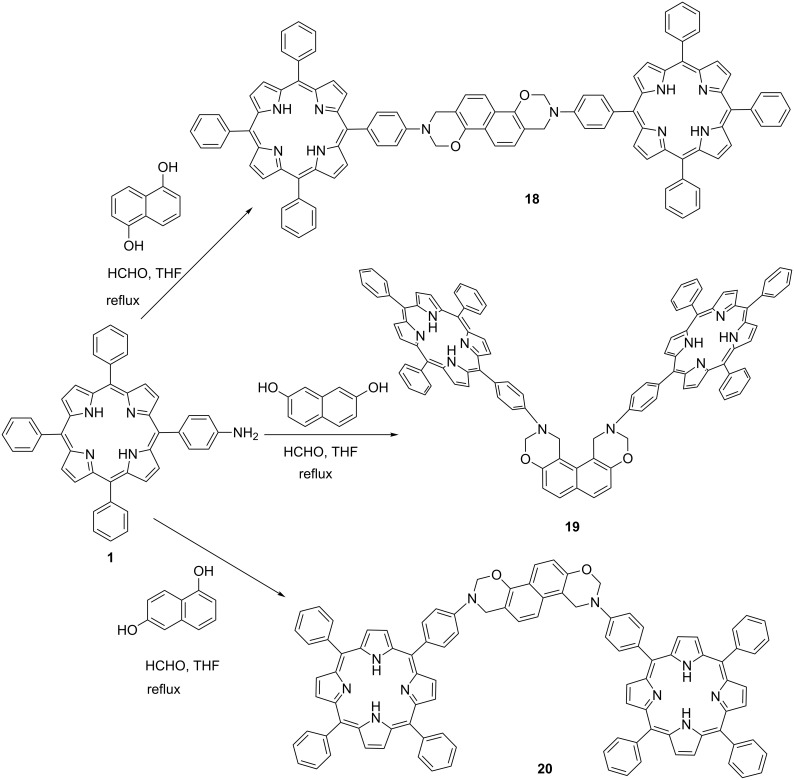
Synthesis of naphtho[*e*]bis(dihydro-1,3-oxazinoporphyrin) derivatives.

In addition, the dimeric naphthoxazinoporphyrins **18–20** were also prepared in moderate yields through one-pot Mannich-type condensation–cyclization reaction of 5-(4-aminophenyl)-10,15,20-triphenylporphyrin (**1**) with α,α- or β,β- or α,β-dihydroxynaphthalenes and formaldehyde in THF under reflux (Scheme 3). Attempts have also been made to prepare the corresponding zinc naphthoxazinoporphyrin dyads, but always impure products were obtained even after repeated column chromatography.

The newly synthesized porphyrins were characterized on the basis of ^1^H NMR, IR, mass and UV–vis data. The ^1^H NMR spectra of all the free-base porphyrins showed a singlet around δ −2.7 ppm corresponding to the internal NH protons. Iminoporphyrins **2** and **3** showed the OH and N=CH protons as singlets around 13.4 and 8.9 ppm, respectively. For the aminoporphyrins **4** and **5**, the hydroxy group and NH protons appeared as broad singlets at 8.4 ppm and 4.3 ppm, respectively. The protons of the CH_2_ group appeared as a singlet at 4.6 ppm. The two characteristic peaks for the dihydro-1,3-oxazinoporphyrins **6**, **7**, **10**, **11** and **14–20** corresponding to N–CH_2_–Ar and N–CH_2_–O groups appeared as two singlets between 4 and 6 ppm. In contrast, the proton NMR spectra of dihydro-1,3-oxazinoporphyrins **8**, **9**, **12** and **13** showed two doublets between 4 and 5 ppm corresponding to one proton each with a coupling constant of 16.5 Hz due to the coupling between two geminal protons of the N–CH_2_–Ar group, and a characteristic singlet around 6–7 ppm for the N–CH–O proton of the oxazine ring. The IR spectra of the porphyrins **2–5** showed a peak around 3400 cm^−1^ corresponding to the hydroxy group. Further, the disappearance of the hydroxy peak in the IR spectra of porphyrins **6–13** confirmed the formation of the oxazine ring. Further, the electronic absorption and emission data of all the synthesized porphyrins are presented in Table 1.

**Table 1:** Electronic absorption and emission data of porphyrins (**2**–**20**).

Compound	Absorption^a^ λ_max_, nm (ε × 10^−4^ , M^−1^ cm^−1^)	Fluorescence^a,b^ (λ_em_/nm)

**2**	421 (34.68), 518 (1.19), 554 (1.03), 593 (0.60), 648 (0.51)	652, 717
**3**	421 (36.60), 517 (1.91), 553 (1.15), 592 (0.82), 647 (0.68)	652, 718
**4**	421 (46.32), 518 (2.20), 553 (1.25), 592 (0.82), 648 (0.68)	653, 717
**5**	421 (44.42), 518 (1.92), 555 (1.02), 592 (0.59), 648 (0.48)	652, 718
**6**	421 (46.82), 518 (2.12), 554 (1.21), 592 (0.78), 649 (0.66)	652, 717
**7**	421 (36.84), 518 (1.90), 554 (1.12), 592 (0.78), 648 (0.66)	652, 717
**8**	421 (52.42), 518 (2.11), 553 (1.36), 592 (0.85), 648 (0.72)	652, 718
**9**	421 (37.75), 518 (1.83), 553 (1.05), 592 (0.72), 647 (0.65)	653, 718
**10**	426 (42.69), 556 (1.81), 597 (0.77)	603, 652
**11**	426 (54.53), 555 (2.26), 597 (0.94)	604, 654
**12**	426 (44.70), 556 (1.97), 597 (0.88)	603, 654
**13**	426 (52.75), 555 (2.35), 597 (1.01)	603, 652
**14**	421 (44.63), 518 (1.90), 554 (1.03), 593 (0.60), 648 (0.51)	654, 717
**15**	426 (41.44), 556 (1.93), 597 (0.90)	605, 655
**16**	421 (44.57), 518 (2.11), 555 (1.22), 592 (0.79), 648 (0.69)	652, 718
**17**	426 (47.83), 556 (2.27), 597 (1.02)	603, 652
**18**	421 (49.94), 518 (2.43), 554 (1.39), 593 (0.79), 649 (0.59)	654, 719
**19**	421 (53.58), 518 (2.59), 555 (1.47), 592 (0.89), 648 (0.74)	654, 718
**20**	421 (46.01), 518 (2.79), 554 (1.68), 592 (1.17), 650 (1.09)	654, 720

^a^Absorption and emission data were measured for CHCl_3_ solutions of porphyrins at 298 K. ^b^Excitation wavelength for the emission data is 420 nm.

**Figure 1:**
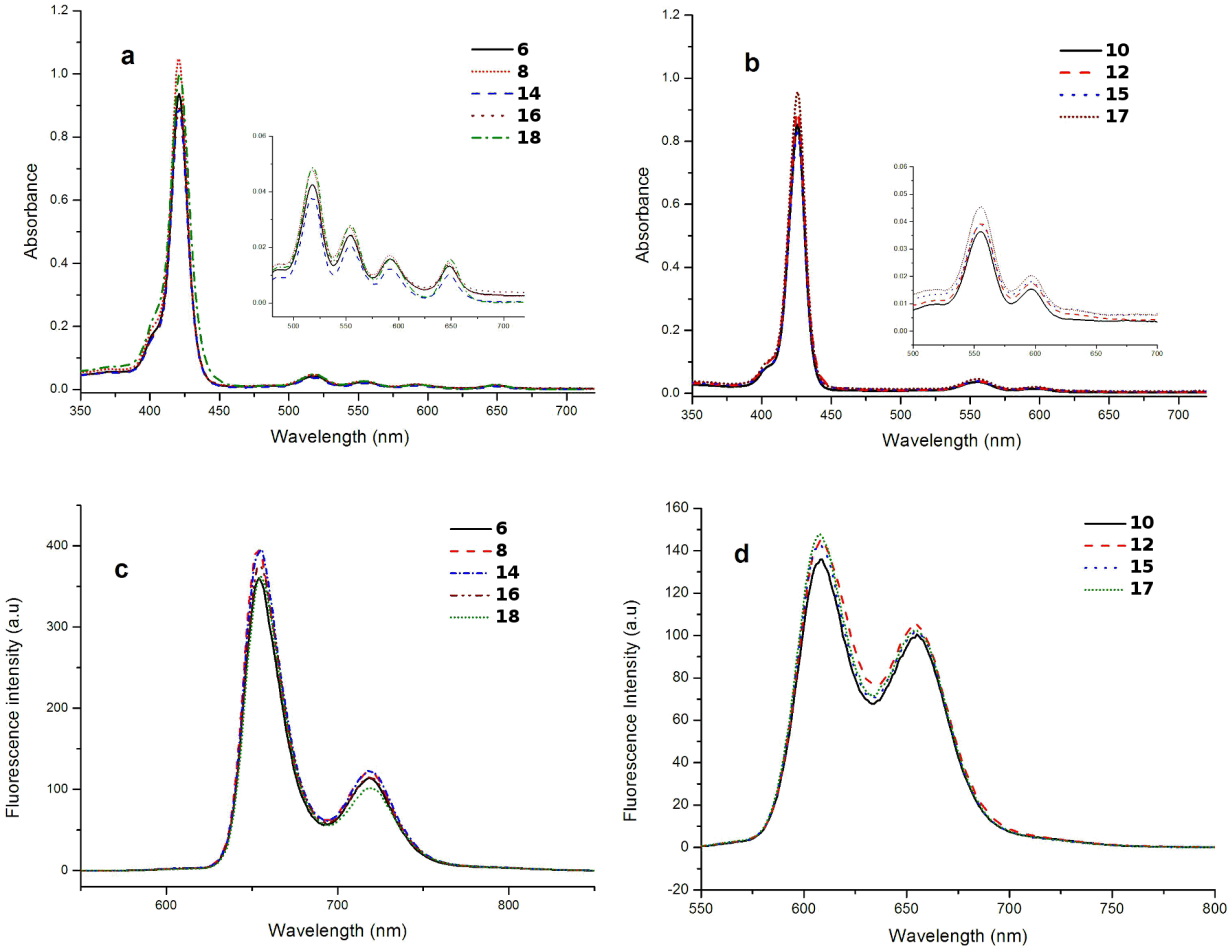
(a) Electronic absorption spectra of free-base porphyrins **6, 8, 14, 16** and **18** in CHCl_3_ at 298 K. (b) Electronic absorption spectra of zinc porphyrins **10, 12, 15** and **17** in CHCl_3_ at 298 K. Inset in both (a) and (b) shows the Q bands. (c) Fluorescence spectra of free-base porphyrins **6, 8, 14, 16** and **18** in CHCl_3_ (2 × 10^−6^ mol L^−1^) at 298 K, λ_ex_ = 420 nm. (d) Fluorescence spectra of zinc porphyrins **10, 12, 15** and **17** in CHCl_3_ (2 × 10^−6^ mol L^−1^) at 298 K, λ_ex_ = 420 nm.

The electronic absorption spectra of all the free-base dihydro-1,3-oxazinoporphyrins exhibited a typical Soret band at 421 nm and four weaker Q bands at ~518, 553, 592 and 648 nm. In contrast, the zinc(II) dihydro-1,3-oxazinoporphyrins showed the Soret band at 426 nm and two Q bands at ~555 and 597 nm. In addition, the newly prepared free-base porphyrins displayed typical emission bands at ~652 and 717 nm, whereas their zinc(II) analogues showed fluorescence bands near 603 and 652 nm. The UV–vis and fluorescence spectra of selected free-base dihydro-1,3-oxazinoporphyrins **6**, **8**, **14**, **16** and **18** and zinc(II) dihydro-1,3-oxazinoporphyrins **10**, **12**, **15** and **17** are shown in Figure 1.

## Conclusion

In summary, we have developed a convenient synthetic protocol for the construction of a new series of dihydro-1,3-oxazinoporphyrins in moderate to good yields. These novel porphyrin-dihydro-1,3-oxazine hybrids may be considered as potential candidates not only for biological evaluations but also for the development of newer π-conjugated molecules for various material applications.

## Supporting Information

File 1 Experimental details and characterization data
