# Supplementary material for: Synthesis of meso-substituted dihydro-1,3-oxazinoporphyrins
Source: Beilstein J Org Chem. 2013 Mar 7;9:496–502. doi: 10.3762/bjoc.9.53 (PMC3628549; doi:10.3762/bjoc.9.53)
Supplement: File 1 — Experimental details and characterization data [file Beilstein_J_Org_Chem-09-496-s001.pdf]

Supporting Information

for

Synthesis of *meso*-substituted dihydro-1,3-oxazinoporphyrins

Satyasheel Sharma and Mahendra Nath*

Address: Department of Chemistry, University of Delhi, Delhi 110 007, India

Email: Mahendra Nath* - mnath@chemistry.du.ac.in

* Corresponding author

Experimental details and characterization data

General:

The chemicals used in the synthesis were purchased either from Sigma-Aldrich or from Merck. The progress of reactions was monitored by thin-layer chromatography (TLC) on silica gel 60 F_{254} (precoated aluminium sheets). The products were purified by column chromatography using activated neutral aluminium oxide (Brokmann grade I–II, Merck) or silica gel 60–120 mesh. ^{1}H NMR spectra were recorded on Brucker 300 or Jeol ECX 400P (400 MHz) NMR spectrometer. Chemical shifts are expressed in parts per million (ppm) relative to tetramethylsilane (TMS; 0 ppm) as an internal standard. Coupling constants *J* are reported in hertz (Hz). Elemental analysis for all the compounds was performed on an Elementar Analysensysteme GmbH VarioEL V3.00. Infrared spectra were recorded on Perkin Elmer IR spectrometer and absorption maxima (υ_{max}) are given in cm^{-1}. UV–vis spectra were recorded on

an Analytik Jena Specord 250 UV–vis spectrophotometer using $CHCl_3$ as a solvent. Fluorescence spectra were recorded on Varian Cary Eclipse fluorescence spectrophotometer. The melting points were determined in open capillary tubes on Buchi M-560 melting point apparatus and are uncorrected.

Synthesis of 5-[4-((2-hydroxybenzylidene)amino)phenyl]-10,15,20-triphenyl-porphyrins 2 and 3

To a solution of 5-(4-aminophenyl)-10,15,20-triphenylporphyrin (**1**, 50 mg, 0.079 mmol) and salicylaldehyde or 5-chlorosalicylaldehyde (0.12 mmol) in toluene (10 mL), $La(OTf)_3$ (9 mg, 0.016 mmol) was added at room temperature. The reaction mixture was stirred under reflux for 8 h. The reaction mixture was allowed to cool at 25 °C and filtered through a cotton plug. The filtrate was evaporated under reduced pressure to produce a purple solid, which on crystallization with chloroform/methanol mixture (1:1) affords a sufficiently pure product.

5-[4-((2-hydroxybenzylidene)amino)phenyl]-10,15,20-triphenylporphyrin (2) [1]

Purple solid; yield: 90%; mp >300 °C; ^{1}H NMR (300 MHz, $CDCl_3$) δ: 13.48 (s, 1H, OH), 9.00–8.86 (m, 9H, CH and *β*-pyrrolic H), 8.30–8.24 (m, 8H, *meso*-ArH), 7.78–7.69 (m, 11H, *meso*-ArH), 7.56 (d, *J* = 6.9 Hz, 1H, ArH), 7.48 (t, *J* = 7.5 Hz, 1H, ArH), 7.14 (d, *J* = 8.1 Hz, 1H, ArH), 7.04 (t, *J* = 7.2 Hz, 1H, ArH), −2.75 (s, 2H, internal NH) ppm.

5-[4-((5-Chloro-2-hydroxy-1-benzylidene)amino)phenyl]-10,15,20-triphenyl-porphyrin (3).

Purple solid; yield: 86%; mp >300 °C; UV λ_{max} ($\varepsilon \times 10^{-4}$, M^{-1} cm^{-1}): 421 (36.60), 517 (1.91), 553 (1.15), 592 (0.82), 647 (0.68) nm; λ_{Em} ($CHCl_3$; λ_{Ex} 420 nm): 652, 718 nm; IR (Film) ν_{max}/cm^{-1}: 3440, 3317, 1618, 1597, 1473, 1342, 1251, 969, 799, 700; ^{1}H NMR (400 MHz, $CDCl_3$) δ: 13.45 (s, 1H, OH), 8.91–8.86 (m, 9H, CH and *β*-pyrrolic H), 8.28 (d, *J* = 8.80 Hz, 2H,

meso-ArH), 8.23–8.21 (m, 6H, *meso*-ArH), 7.78–7.73 (m, 9H, *meso*-ArH), 7.67 (d, *J* = 8.08 Hz, 2H, *meso*-ArH), 7.52 (d, *J* = 2.2 Hz, 1H, ArH), 7.40 (dd, J_1 = 8.79 Hz, J_2 = 2.2 Hz, 1H, ArH), 7.07 (d, *J* = 8.79 Hz, 1H, ArH), −2.76 (s, 2H, internal NH) ppm. ESI-MS: *m*/*z* = 768 $(M + H)^+$; Anal. calcd for $C_{51}H_{34}ClN_5O{\cdot}H_2O$: C, 77.90; H, 4.61; N, 8.91; found: C, 77.80; H, 4.83; N, 8.67.

Synthesis of 5-[4-((2-hydroxybenzyl)amino)phenyl)-10,15,20-triphenylporphyrins 4 and 5

To a solution of iminoporphyrin **2** or **3** (0.065 mmol) in chloroform (10 mL), a solution of $NaBH_4$ (15 mg, 0.190 mmol) in methanol (1 mL) was added, and the reaction mixture was stirred at room temperature for 15 min. After completion of the reaction, the mixture was washed with water and organic layer was dried over anhydrous Na_2SO_4. The organic layer was evaporated under reduced pressure. Thus, the crude product obtained was purified over silica gel column using 80% $CHCl_3$ in hexane as eluent.

5-[4-((2-Hydroxybenzyl)amino)phenyl)]-10,15,20-triphenylporphyrin (4)

Purple solid; yield: 90%; mp >300 °C; UV λ_{max} ($\varepsilon \times 10^{-4}$, M^{-1} cm^{-1}): 421 (46.32), 518 (2.20), 553 (1.25), 592 (0.82), 648 (0.68) nm; λ_{Em} ($CHCl_3$; λ_{Ex} 420 nm): 653, 717 nm; IR (Film) ν_{max}/cm^{-1}: 3492, 3381, 1630, 1595, 1559, 1517, 1473, 1441, 1345, 1252, 968, 800, 731, 701; ^1H NMR (300 MHz, $CDCl_3$) δ: 8.88 (s, 2H, *β*-pyrrolic H), 8.83 (s, 6H, *β*-pyrrolic H), 8.34 (brs, 1H, OH), 8.22 (s, 6H, *meso*-ArH), 8.06 (d, *J* = 7.8 Hz, 2H, *meso*-ArH), 7.76 (s, 9H, *meso*-ArH), 7.31 (d, *J* = 6.6 Hz, 2H, *meso*-ArH), 7.18 (d, *J* = 8.1 Hz, 2H, ArH), 7.01–6.93 (m, 2H, ArH), 4.66 (s, 2H, CH_2), 4.32 (brs, 1H, NH), −2.76 (s, 2H, internal NH) ppm. ESI-MS: *m*/*z* = 736 $(M + H)^+$; Anal. calcd for $C_{51}H_{37}N_5O$: C, 83.24; H, 5.07; N, 9.52; found: C, 83.44; H, 4.90; N, 9.26.

5-[4-((5-Chloro-2-hydroxybenzyl)amino)phenyl)]-10,15,20-triphenylporphyrin (5).

Purple solid; yield: 90%; mp >300 °C; UV λ_{max} ($\varepsilon \times 10^{-4}$, M^{-1} cm^{-1}): 421 (44.42), 518 (1.92), 555 (1.02), 592 (0.59), 648 (0.48) nm; λ_{Em} ($CHCl_3$; λ_{Ex} 420 nm): 652, 718 nm; IR (Film) ν_{max}/cm^{-1}: 3490, 3376, 1620, 1597, 1560, 1473, 1440, 1345, 1252, 968, 800, 701; ^1H NMR (400 MHz, $CDCl_3$) δ: 8.87–8.83 (m, 8H, *β*-pyrrolic H), 8.47 (brs, 1H, OH), 8.21 (d, *J* = 7.32 Hz, 6H, *meso*-ArH), 8.04 (d, *J* = 8.05 Hz, 2H, *meso*-ArH), 7.78–7.71 (m, 9H, *meso*-ArH), 7.25 (d, *J* = 2.20 Hz, 1H, ArH), 7.22 (dd, J_1 = 8.42 Hz, J_2 = 2.2 Hz, 1H, ArH), 7.10 (d, *J* = 8.05 Hz, 2H, *meso*-ArH), 6.88 (d, *J* = 8.05 Hz, 1H, ArH), 4.56 (s, 2H, CH_2), 4.24 (brs, 1H, NH), −2.77 (s, 2H, internal NH) ppm. ESI-MS: *m/z* = 770 $(M + H)^+$; Anal. calcd for $C_{51}H_{36}ClN_5O$: C, 79.52; H, 4.71; N, 9.09; found: C, 79.83; H, 4.88; N, 8.89.

Synthesis of dihydro-1,3-benzoxazinoporphyrins (6–9)

To a solution of aminoporphyrin (**4** or **5**; 0.045 mmol) in THF (10 mL), 37% aq formalin solution (30 µL) or *p*-trifluorotolualdehyde (100 µL) was added and the reaction mixture was heated under reflux (14 h for **6**–**7**, 24 h for **8** and 40 h for **9**). After completion of the reaction, solvent was removed under reduced pressure. Thus, the residue obtained was dissolved in chloroform and washed thoroughly with water. Organic layer was dried over anhydrous sodium sulphate and evaporated under reduced pressure. The title compounds were crystallized by using 50% methanol in chloroform.

5-[4-(3,4-Dihydro-2*H*-benzo[*e*][1,3]oxazin-3-yl)phenyl]-10,15,20-triphenylporphyrin (6)

Purple solid; yield: 85%; mp >300 °C; UV λ_{max} ($\varepsilon \times 10^{-4}$, M^{-1} cm^{-1}): 421 (46.82), 518 (2.12), 554 (1.21), 592 (0.78), 649 (0.66) nm; λ_{Em} ($CHCl_3$; λ_{Ex} 420 nm): 652, 717 nm; IR (Film) ν_{max}/cm^{-1}: 3318, 1596, 1560, 1473, 1440, 1350, 1217, 1179, 1071, 965, 798, 731, 701; ^1H NMR

(300 MHz, $CDCl_3$) δ: 8.89 (d, *J* = 4.8 Hz, 2H, *β*-pyrrolic H), 8.82 (s, 6H, *β*-pyrrolic H), 8.21 (d, *J* = 5.4 Hz, 6H, *meso*-ArH), 8.10 (d, *J* = 8.4 Hz, 2H, *meso*-ArH), 7.74 (d, *J* = 6.6 Hz, 9H, *meso*-ArH), 7.47 (d, *J* = 8.4 Hz, 2H, *meso*-ArH), 7.19 (d, *J* = 8.1 Hz, 2H, ArH), 7.03–6.95 (m, 2H, ArH), 5.65 (s, 2H, O-CH_2-N), 4.93 (s, 2H, N-CH_2-Ar), −2.78 (s, 2H, internal NH) ppm. ESI-MS: *m*/*z* = 748 $(M + H)^+$; Anal. calcd for $C_{52}H_{37}N_5O$: C, 83.51; H, 4.99; N, 9.36; found: C, 83.42; H, 4.78; N, 9.52.

5-[4-(6-Chloro-3,4-dihydro-2*H*-benzo[*e*][1,3]oxazin-3-yl)phenyl]-10,15,20-triphenyl-porphyrin (7)

Purple solid; yield: 78%; mp >300 °C; UV λ_{max} ($\varepsilon \times 10^{-4}$, M^{-1} cm^{-1}): 421 (36.84), 518 (1.90), 554 (1.12), 592 (0.78), 648 (0.66) nm; λ_{Em} ($CHCl_3$; λ_{Ex} 420 nm): 652, 717 nm; IR (Film) ν_{max}/cm^{-1}: 3316, 1597, 1559, 1473, 1440, 1353, 1217, 1072, 969, 799, 701; ^1H NMR (300 MHz, $CDCl_3$) δ: 8.88–8.83 (m, 8H, *β*-pyrrolic H), 8.20 (d, *J* = 5.7 Hz, 6H, *meso*-ArH), 8.10 (d, *J* = 8.1 Hz, 2H, *meso*-ArH), 7.74 (d, *J* = 6.3 Hz, 9H, *meso*-ArH), 7.43 (d, *J* = 8.4 Hz, 2H, *meso*-ArH), 7.18–7.15 (m, 2H, ArH), 6.88 (d, *J* = 8.4 Hz, 1H, ArH), 5.60 (s, 2H, O-CH_2-N), 4.86 (s, 2H, N-CH_2-Ar), −2.77 (s, 2H, internal NH) ppm. ESI-MS: *m*/*z* = 782 $(M + H)^+$; Anal. calcd for $C_{52}H_{36}ClN_5O$: C, 79.83; H, 4.64; N, 8.95; found: C, 79.58; H, 4.71; N, 9.21.

5-{4-[2-(4-Trifluoromethylphenyl)-3,4-dihydro-2*H*-benzo[*e*][1,3]oxazin-3-yl]-phenyl}-10,15,20-triphenylporphyrins (8)

Purple solid; yield: 75%; mp >300 °C; UV λ_{max} ($\varepsilon \times 10^{-4}$, M^{-1} cm^{-1}): 421 (52.42), 518 (2.11), 553 (1.36), 592 (0.85), 648 (0.72) nm; λ_{Em} ($CHCl_3$; λ_{Ex} 420 nm): 652, 718 nm. IR (Film) ν_{max}/cm^{-1}: 3318, 1596, 1560, 1473, 1440, 1350, 1217, 965, 798, 731, 701; ^1H NMR (300 MHz, $CDCl_3$) δ: 8.80 (s, 2H, *β*-pyrrolic H), 8.76 (s, 6H, *β*-pyrrolic H), 8.12 (s, 6H, *meso*-ArH), 8.04 (d, *J* = 7.8 Hz, 2H, *meso*-ArH), 7.78 (d, *J* = 7.8 Hz, 2H, *meso*-ArH), 7.68 (s, 11H, *meso*-ArH and

ArH), 7.77 (d, *J* = 7.8 Hz, 2H, ArH), 7.17 (s, 1H, ArH), 7.06 (d, *J* = 7.8 Hz, 1H, ArH), 6.95–6.89 (m, 3H, ArH and N-CH-O), 4.61 (d, *J* = 16.5 Hz, 1H, N-CH-Ar), 4.44 (d, *J* = 16.5 Hz, 1H, N-CH-Ar), −2.85 (s, 2H, internal NH) ppm. ESI-MS: *m*/*z* = 892 $(M + H)^+$; Anal. calcd for $C_{59}H_{40}F_3N_5O{\cdot}H_2O$: C, 77.87; H, 4.65; N, 7.70; found: C, 77.92; H, 4.74; N, 7.52.

5-{4-[6-Chloro-2-(4-trifluoromethylphenyl)-3,4-dihydro-2*H*-benzo[*e*][1,3]oxazin-3-yl]phenyl}-10,15,20-triphenylporphyrin (9)

Purple solid; yield: 70%; mp >300 °C; UV λ_{max} ($\varepsilon \times 10^{-4}$, M^{-1} cm^{-1}): 421 (37.75), 518 (1.83), 553 (1.05), 592 (0.72), 647 (0.65) nm; λ_{Em} ($CHCl_3$; λ_{Ex} 420 nm): 653, 718 nm; IR (Film) ν_{max}/cm^{-1}: 3318, 1597, 1560, 1473, 1230, 1187, 1101, 969, 800, 752, 700; ^1H NMR (300 MHz, $CDCl_3$) δ: 8.83 (s, 8H, *β*-pyrrolic H), 8.20 (s, 6H, *meso*-ArH), 8.13 (d, *J* = 6.9 Hz, 2H, *meso*-ArH), 7.81–7.75 (m, 13H, *meso*-ArH and ArH), 7.53 (d, *J* = 7.2 Hz, 2H, ArH), 7.25 (s, 1H, ArH), 7.15–7.03 (m, 2H, ArH), 6.96 (s, 1H, N-CH-O), 4.64 (d, *J* = 16.2 Hz, 1H, N-CH-Ar), 4.47 (d, *J* = 15.6 Hz, 1H, N-CH-Ar), −2.77 (s, 2H, internal NH) ppm; ESI-MS: *m*/*z* = 926 $(M + H)^+$; Anal. calcd for $C_{59}H_{39}ClF_3N_5O$: C, 76.49; H, 4.24; N, 7.56; found: C, 76.42; H, 4.36; N, 7.84.

Synthesis of dihydro-1,3-naphthoxazinoporphyrins 14 and 16

To a solution of 5-(4-aminophenyl)-10,15,20-triphenylporphyrin (**1**, 50 mg, 0.079 mmol) and *β*-naphthol or *α*-naphthol (15 mg, 0.104 mmol) in THF (10 mL), 37% aq formalin solution (150 µL) was added. The reaction mixture was heated under reflux at 80 °C for 16 h. After completion of the reaction, THF was removed under reduced pressure. Thus, the residue obtained was dissolved in chloroform and washed thoroughly with water. The organic layer was dried over anhydrous sodium sulfate and evaporated under reduced pressure. The crude product was loaded on silica gel column and the pure product (**14** or **16**) was obtained after eluting the column with chloroform.

5-[4-(2,3-Dihydro-1*H*-naphtho[1,2-*e*][1,3]oxazin-2-yl)phenyl]-10,15,20-triphenylporphyrin (14)

Purple solid; yield: 85%; mp >300 °C; UV λ_{max} ($\varepsilon \times 10^{-4}$, M^{-1} cm^{-1}): 421 (44.63), 518 (1.90), 554 (1.03), 593 (0.60), 648 (0.51) nm; λ_{Em} ($CHCl_3$; λ_{Ex} 420 nm): 654, 717 nm; IR (Film) ν_{max}/cm^{-1}: 3317, 1598, 1542, 1508, 1473, 1438, 1230, 1187, 965, 801, 747, 701; ^1H NMR (300 MHz, $CDCl_3$) δ: 8.88 (d, *J* = 4.5 Hz, 2H, *β*-pyrrolic H), 8.82 (s, 6H, *β*-pyrrolic H), 8.20 (d, *J* = 6.0 Hz, 6H, *meso*-ArH), 8.10 (d, *J* = 8.1 Hz, 2H, *meso*-ArH), 7.83 (d, *J* = 8.4 Hz, 2H, *meso*-ArH), 7.74–7.72 (m, 9H, *meso*-ArH), 7.59–7.50 (m, 4H, ArH), 7.42 (t, *J* = 7.5 Hz, 1H, ArH), 7.19 (d, *J* = 9.0 Hz, 1H, ArH), 5.70 (s, 2H, N-CH_2-O), 5.24 (s, 2H, N-CH_2-Ar), −2.78 (s, 2H, internal NH) ppm; ESI-MS: *m*/*z* = 798 $(M + H)^+$; Anal. calcd for $C_{56}H_{39}N_5O$: C, 84.29; H, 4.93; N, 8.78; found: C, 84.22; H, 4.97; N, 8.52.

5-[4-(3,4-Dihydro-2*H*-naphtho[2,1-*e*][1,3]oxazin-3-yl)phenyl]-10,15,20-triphenylporphyrin (16)

Purple solid; yield: 75%; mp >300 °C; UV λ_{max} ($\varepsilon \times 10^{-4}$, M^{-1} cm^{-1}): 421 (44.57), 518 (2.11), 555 (1.22), 592 (0.79), 648 (0.69) nm; λ_{Em} ($CHCl_3$; λ_{Ex} 420 nm): 652, 718 nm; IR (Film) ν_{max}/cm^{-1}: 3316, 1597, 1560, 1473, 1232, 1187, 1101, 969, 801, 753, 700; ^1H NMR (300 MHz, $CDCl_3$) δ: 8.88 (d, *J* = 4.8 Hz, 2H, *β*-pyrrolic H), 8.82–8.79 (m, 6H, *β*-pyrrolic H), 8.20 (d, *J* = 7.2 Hz, 6H, *meso*-ArH), 8.10 (d, *J* = 8.4 Hz, 2H, *meso*-ArH), 7.83–7.76 (m, 11H, *meso*-ArH), 7.54–7.41 (m, 5H, ArH), 7.26 (d, *J* = 7.8 Hz, 1H, ArH), 5.82 (s, 2H, N-CH_2-O), 5.03 (s, 2H, N-CH_2-Ar), −2.78 (s, 2H, internal NH) ppm. ESI-MS: *m*/*z* = 798 $(M + H)^+$; Anal. calcd for $C_{56}H_{39}N_5O \cdot 0.5H_2O$: C, 83.35; H, 5.00; N, 8.68; found: C, 82.97; H, 4.96; N, 8.32.

Synthesis of zinc(II) dihydro-1,3-oxazinoporphyrins (10–13, 15 and 17)

To a solution of the corresponding free-base dihydro-1,3-oxazinoporphyrin (0.04 mmol) in chloroform (10 mL), a solution of zinc acetate (10 mg, 0.05 mmol) in methanol (1 mL) was added. The reaction mixture was stirred at room temperature for 30 min. After completion of the reaction, the mixture was washed with water, and the $CHCl_3$ layer was dried over anhydrous Na_2SO_4. The organic layer was evaporated under reduced pressure to afford the crude product, which was purified by recrystallization using 50% methanol in chloroform.

Zinc(II) 5-[4-(3,4-dihydro-2*H*-benzo[*e*][1,3]oxazin-3-yl)phenyl]-10,15,20-triphenyl-porphyrin (10)

Purple solid; yield: 90%; mp >300 °C; UV λ_{max} ($\varepsilon \times 10^{-4}$, M^{-1} cm^{-1}): 426 (42.69), 556 (1.81), 597 (0.77) nm; λ_{Em} ($CHCl_3$; λ_{Ex} 420 nm): 603, 652 nm; IR (Film) ν_{max}/cm^{-1}: 1598, 1559, 1453, 1351, 1242, 1076, 1008, 912, 794, 751, 700; ^1H NMR (400 MHz, $CDCl_3$) δ: 8.83 (s, 2H, *β*-pyrrolic H), 8.77 (s, 6H, *β*-pyrrolic H), 8.13 (s, 6H, *meso*-ArH), 8.02 (s, 2H, *meso*-ArH), 7.98 (s, 2H, *meso*-ArH), 7.72 (s, 9H, *meso*-ArH), 7.45 (d, *J* = 6.6 Hz, 2H, ArH), 6.93–6.87 (m, 2H, ArH), 5.64 (s, 2H, N-CH_2-O), 4.89 (s, 2H, N-CH_2-Ar) ppm. ESI-MS: *m*/*z* = 810 $(M + H)^+$; Anal. calcd for $C_{52}H_{35}N_5OZn\cdot1.2H_2O$: C, 74.99; H, 4.53; N, 8.41; found: C, 75.12; H, 4.77; N, 8.27.

Zinc(II) 5-[4-(6-chloro-3,4-dihydro-2*H*-benzo[*e*][1,3]oxazin-3-yl)phenyl]-10,15,20-triphenylporphyrin (11)

Purple solid; yield: 88%; mp >300 °C; UV λ_{max} ($\varepsilon \times 10^{-4}$, M^{-1} cm^{-1}): 426 (54.53), 555 (2.26), 597 (0.94) nm; λ_{Em} ($CHCl_3$; λ_{Ex} 420 nm): 604, 654 nm; IR (Film) ν_{max}/cm^{-1}: 1597, 1560, 1472, 1351, 1260, 1180, 1075, 1009, 850, 797, 754, 700; ^1H NMR (400 MHz, $CDCl_3$) δ: 8.98–8.93 (m, 8H, *β*-pyrrolic H), 8.21 (d, *J* = 5.8 Hz, 6H, *meso*-ArH), 8.10 (d, *J* = 8.8 Hz, 2H, *meso*-ArH), 7.75–7.73 (m, 11H, *meso*-ArH), 7.40 (d, *J* = 8.4 Hz, 2H, ArH), 7.15 (s, 1H, ArH), 5.54 (s, 2H, N-

CH_2-O), 4.81 (s, 2H, N-CH_2-Ar) ppm; ESI-MS: *m*/*z* = 844 $(M + H)^+$; Anal. calcd for $C_{52}H_{34}ClN_5OZn\cdot2H_2O$: C, 70.83; H, 4.34; N, 7.94; found: C, 71.14; H, 4.52; N, 8.22.

Zinc(II) 5-{4-[2-(4-trifluoromethylphenyl)-3,4-dihydro-2*H*-benzo[*e*][1,3]oxazin-3-yl]phenyl}-10,15,20-triphenylporphyrin (12)

Purple solid; yield: 90%; mp >300 °C; UV λ_{max} ($\varepsilon \times 10^{-4}$, M^{-1} cm^{-1}): 426 (44.70), 556 (1.97), 597 (0.88) nm; λ_{Em} ($CHCl_3$; λ_{Ex} 420 nm): 603, 654 nm; IR (Film) ν_{max}/cm^{-1}: 1598, 1560, 1441, 1351, 1261, 1214, 1178, 1074, 1009, 968, 799, 753, 700; ^1H NMR (400 MHz, $CDCl_3$) δ: 8.96–8.91 (m, 8H, *β*-pyrrolic H), 8.20 (d, *J* = 6.6 Hz, 8H, *meso*-ArH), 8.02 (d, *J* = 8.0 Hz, 2H, ArH), 7.74–7.72 (m, 11H, *meso*-ArH), 7.24–7.22 (m, 3H, ArH), 7. 04 (d, *J* = 7.2 Hz, 2H, ArH), 6.91–6.83 (m, 2H, ArH and N-CH-O), 4.48 (d, *J* = 16.5 Hz, 2H, N-CH_2-Ar) ppm; ESI-MS: *m*/*z* = 954 $(M + H)^+$; Anal. calcd for $C_{59}H_{38}F_3N_5OZn\cdot1.5H_2O$: C, 72.13; H, 4.21; N, 7.13; found: C, 72.21; H, 4.44; N, 7.41.

Zinc(II) 5-{4-[6-chloro-2-(4-trifluoromethylphenyl)-3,4-dihydro-2*H*-benzo[*e*][1,3]-oxazin-3-yl]phenyl}-10,15,20-triphenylporphyrin (13)

Purple solid; yield: 90%; mp >300 °C; UV λ_{max} ($\varepsilon \times 10^{-4}$, M^{-1} cm^{-1}): 426 (52.75), 555 (2.35), 597 (1.01) nm; λ_{Em} ($CHCl_3$; λ_{Ex} 420 nm): 603, 652 nm; IR (Film) ν_{max}/cm^{-1}: 1597, 1560, 1472, 1351, 1260, 1215, 1180, 1075, 1009, 850, 797, 754, 700; ^1H NMR (400 MHz, $CDCl_3$) δ: 8.98–8.93 (m, 8H, *β*-pyrrolic H), 8.21 (d, *J* = 7.2 Hz, 6H, *meso*-ArH), 8.13 (d, *J* = 8.0 Hz, 2H, *meso*-ArH), 7.82 (d, *J* = 8.0 Hz, 2H, ArH), 7.75–7.70 (m, 11H, *meso*-ArH), 7.53 (d, *J* = 8.0 Hz, 2H, ArH), 7.23–7.21 (m, 1H, ArH), 7.07 (s, 1H, ArH), 7.05–7.03 (m, 1H, ArH), 6.94 (s, 1H, N-CH-O), 4.65 (d, *J* = 17.0 Hz, 1H, N-CH-Ar), 4.48 (d, *J* = 16.8 Hz, 1H, N-CH-Ar) ppm.; ESI-MS: *m*/*z* = 988 $(M + H)^+$; Anal. calcd for $C_{59}H_{37}ClF_3N_5OZn\cdot2H_2O$: C, 69.08; H, 4.03; N, 6.83; found: C, 68.92; H, 4.27; N, 7.17.

Zinc(II) 5-[4-(2,3-dihydro-1*H*-naphtho[1,2-*e*][1,3]oxazin-2-yl)phenyl]-10,15,20-triphenylporphyrin (15)

Purple solid; yield: 90%; mp >300 °C; UV λ_{max} ($\varepsilon \times 10^{-4}$, M^{-1} cm^{-1}): 426 (41.44), 556 (1.93), 597 (0.90) nm; λ_{Em} ($CHCl_3$; λ_{Ex} 420 nm): 605, 655 nm; IR (Film) ν_{max}/cm^{-1}: 1598, 1560, 1473, 1351, 1305, 1218, 1160, 1073, 1009, 796, 752, 701; ^1H NMR (400 MHz, $CDCl_3$) δ: 8.96 (d, *J* = 4.39 Hz, 2H, *β*-pyrrolic H), 8.91–8.89 (m, 6H, *β*-pyrrolic H), 8.19 (d, *J* = 6.59 Hz, 6H, *meso*-ArH), 8.07 (d, *J* = 8.05 Hz, 2H, *meso*-ArH), 7.83–7.70 (m, 11H, *meso*-ArH), 7.58–7.39 (m, 4H, ArH), 7.16 (d, *J* = 9.5 Hz, 2H, ArH), 5.60 (s, 2H, N-CH_2-O), 5.13 (s, 2H, N-CH_2-Ar) ppm; ESI-MS: *m*/*z* = 860 $(M + H)^+$; Anal. calcd for $C_{56}H_{37}N_5OZn.2H_2O$: C, 74.95; H, 4.61; N, 7.80; found: C, 75.15; H, 4.88; N, 8.01.

Zinc(II) 5-[4-(3,4-dihydro-2*H*-naphtho[2,1-*e*][1,3]oxazin-3-yl)phenyl]-10,15,20-triphenylporphyrin (17)

Purple solid; yield: 92%; mp >300 °C; UV λ_{max} ($\varepsilon \times 10^{-4}$, M^{-1} cm^{-1}): 426 (47.83), 556 (2.27), 597 (1.02) nm; λ_{Em} ($CHCl_3$; λ_{Ex} 420 nm): 603, 652 nm; IR (Film) ν_{max}/cm^{-1}: 1598, 1559, 1453, 1351, 1242, 1076, 1008, 850, 796, 752, 700; ^1H NMR (400 MHz, $CDCl_3$) δ: 8.89 (d, *J* = 4.39 Hz, 2H, *β*-pyrrolic H), 8.85 (s, 4H, *β*-pyrrolic H), 8.82 (d, *J* = 4.39 Hz, 2H, *β*-pyrrolic H), 8.14−8.12 (m, 6H, *meso*-ArH), 8.00 (d, *J* = 8.05 Hz, 2H, *meso*-ArH), 7.68−7.63 (m, 11H, *meso*-ArH), 7.42−7.34 (m, 6H, ArH), 5.61 (s, 2H, N-CH_2-O), 4.82 (s, 2H, N-CH_2-Ar) ppm; ESI-MS: *m*/*z* = 860 $(M + H)^+$; Anal. calcd for $C_{56}H_{37}N_5OZn\cdot H_2O$: C, 76.49; H, 4.47; N, 7.96; found: C, 76.82; H, 4.68; N, 8.04.

Synthesis of naphtho[e]bis(dihydro-1,3-oxazinoporphyrin) derivatives (18–20)

To a solution of 5-(4-aminophenyl)-10,15,20-triphenylporphyrin (**1**, 50 mg, 0.079 mmol) and the corresponding dihydroxynaphthalene (7 mg, 0.14 mmol) in THF (10 mL), 37% formalin solution

(200 µL) was added. The reaction mixture was heated under reflux at 80 °C for 24–30 h. After completion of the reaction, the solvent was removed under reduced pressure. Thus, the residue obtained was dissolved in chloroform and washed thoroughly with water. The organic layer was dried over anhydrous sodium sulfate and evaporated under reduced pressure. The desired products were purified by crystallization using chloroform-methanol (1:1) mixture.

Dihydro-1,3-naphthoxazinoporphyrin dyad (18)

Purple solid; yield: 45%; mp >300 °C; UV λ_{max} ($\varepsilon \times 10^{-4}$, M^{-1} cm^{-1}): 421 (49.94), 518 (2.43), 554 (1.39), 593 (0.79), 649 (0.59) nm; λ_{Em} ($CHCl_3$; λ_{Ex} 420 nm): 654, 719 nm; IR (Film) ν_{max}/cm^{-1}: 3318, 1600, 1521, 1476, 1440, 1347, 1223, 1181, 965, 801, 751, 702; ^1H NMR (300 MHz, $CDCl_3$) δ: 8.89–8.80 (m, 16H, *β*-pyrrolic H), 8.19–8.09 (m, 16H, *meso*-ArH) 7.91–7.70 (m, 20H, *meso*-ArH) 7.52 (d, *J* = 7.2 Hz, 4H, *meso*-ArH and ArH), 7.29 (d, *J* = 9 Hz, 2H, ArH), 5.81 (s, 4H, N-CH_2-O), 5.02 (s, 4H, N-CH_2-Ar), −2.77 (s, 4H, internal NH) ppm; ESI-MS: *m*/*z* = 1467.5 $(M + H)^+$; Anal. calcd for $C_{102}H_{70}N_{10}O_2 \cdot 2H_2O$: C, 81.47; H, 4.96; N, 9.31; found: C, 81.23; H, 5.08; N, 9.25.

Dihydro-1,3-naphthoxazinoporphyrin dyad (19)

Purple solid; yield: 40%; mp >300 °C; UV λ_{max} ($\varepsilon \times 10^{-4}$, M^{-1} cm^{-1}): 421 (53.58), 518 (2.59), 555 (1.47), 592 (0.89), 648 (0.74) nm; λ_{Em} ($CHCl_3$; λ_{Ex} 420 nm): 654, 718 nm; IR (Film) ν_{max}/cm^{-1}: 3316, 1597, 1560, 1473, 1232, 1187, 1101, 969, 801, 753, 700; ^1H NMR (300 MHz, $CDCl_3$) δ: 8.96–8.80 (m, 16H, *β*-pyrrolic H), 8.29 (s, 14H, *meso*-ArH), 8.09 (d, *J* = 8.1 Hz, 2H, *meso*-ArH), 7.75–7.61 (m, 22H, *meso*-ArH), 7.49 (d, *J* = 8.1 Hz, 2H, ArH), 6.96–6.88 (m, 2H, ArH), 5.67 (s, 4H, N-CH_2-O), 5.09 (s, 4H, N-CH_2-Ar), −2.78 (s, 4H, internal NH) ppm; ESI-MS: *m*/*z* = 1467.5 $(M + H)^+$; Anal. calcd for $C_{102}H_{70}N_{10}O_2$: C, 83.47; H, 4.81; N, 9.54; found: C, 83.69; H, 5.18; N, 9.33.

Dihydro-1,3-naphthoxazinoporphyrin dyad (20)

Purple solid; yield: 40%; mp >300 °C; UV λ_{max} ($\varepsilon \times 10^{-4}$, M^{-1} cm^{-1}): 421 (46.01), 518 (2.79), 554 (1.68), 592 (1.17), 650 (1.09) nm; λ_{Em} ($CHCl_3$; λ_{Ex} 420 nm): 654, 720 nm; IR (Film) ν_{max}/cm^{-1}: 3316, 1597, 1560, 1473, 1232, 1187, 1101, 969, 801, 753, 700; ^{1}H NMR (300 MHz, $CDCl_3$) δ: 8.88–8.82 (m, 16H, *β*-pyrrolic H), 8.28–8.07 (m, 16H, *meso*-ArH), 7.74 (s, 22H, *meso*-ArH), 7.49 (d, *J* = 8.1 Hz, 2H, ArH), 7.17 (d, *J* = 8.7 Hz, 1H, ArH), 7.06 (d, *J* = 8.7 Hz, 1H, ArH), 5.71 (s, 4H, N-CH_2-O), 4.95 (s, 4H, N-CH_2-Ar), −2.78 (s, 4H, internal NH) ppm; ESI-MS: *m*/*z* = 1467.5 $(M + H)^+$; Anal. calcd for $C_{102}H_{70}N_{10}O_2 \cdot 3H_2O$: C, 80.50; H, 5.03; N, 9.20; found: C, 80.67; H, 5.38; N, 9.03.

Reference:

1. Hayvalı, M.; Gündüz, H.; Gündüz, N.; Kılıç, Z.; Hökelek, T. *J. Mol. Struc.* **2000,** *525*, 215–226. doi: 10.1016/S0022-2860(00)00417-8
